# Tessituras de memórias: o Instituto Fernandes Figueira pelo olhar geracional

**DOI:** 10.1590/S0104-59702024000100003

**Published:** 2024-04-05

**Authors:** Martha Cristina Nunes Moreira, Roberta Falcão Tanabe, Marina Castinheiras Diuana, Anita Silva Paez

**Affiliations:** i Docente Permanente, Programa de Pós-graduação em Saúde da Criança e da Mulher/Instituto Nacional de Saúde da Mulher, da Criança e do Adolescente Fernandes Figueira/Fiocruz. Rio de Janeiro – RJ – Brasil martha.moreira@fiocruz.br; ii Tecnologista em Saúde Pública, Coordenadora do Núcleo Saúde e Brincar/Instituto Nacional de Saúde da Mulher, da Criança e do Adolescente Fernandes Figueira/Fiocruz. Rio de Janeiro – RJ – Brasil roberta.tanabe@fiocruz.br; iii Doutoranda, Programa de Pós-graduação em Saúde da Criança e da Mulher/Instituto Nacional de Saúde da Mulher, da Criança e do Adolescente Fernandes Figueira/Fiocruz. Rio de Janeiro – RJ – Brasil marinacdiuana@gmail.com; iv Tecnologista em Saúde Pública, Coordenadora do Núcleo Saúde e Brincar/Instituto Nacional de Saúde da Mulher, da Criança e do Adolescente Fernandes Figueira/Fiocruz. Rio de Janeiro – RJ – Brasil anita.paez@fiocruz.br

**Keywords:** Memory, Care, Narratives, Generation, Instituto Nacional de Saúde da Mulher, da Criança e do Adolescente Fernandes Figueira (IFF), Memória, Cuidado, Narrativas, Geração, Instituto Nacional de Saúde da Mulher, da Criança e do Adolescente Fernandes Figueira (IFF)

## Abstract

Recuperamos memórias do Instituto Fernandes Figueira via o cuidado que reúne crianças doentes e suas mães. A categoria analítica geração sustenta o argumento do instituto como espaço de experiências e memórias. Interpretamos três fontes de memórias: (1) a pesquisa de Marismary Horsth De Seta com a geração que chegou no instituto na década de 1940; (2) o relatório de atividades do instituto de 1973; (3) três entrevistas com trabalhadores admitidos na década de 1980. Concluímos que o cuidado com as crianças e, por conseguinte, um olhar para as mulheres nessa relação se dão em sintonia com a transição epidemiológica global, complexificando o perfil da atenção do instituto.

## Abstract (en)

Memories of care involving sick children and their mothers at the Fernandes Figueira Institute (Instituto Fernandes Figueira) are retrieved. The analysis using a generational perspective reveals the institute as a space of experiences and memories. Three sources of memories are analysed: (1) the research by Marismary Horsth De Seta with the generation that reached the institute in the 1940s; (2) the institute’s 1973 activity report; (3) three interviews with workers admitted in the 1980s. It is concluded that care for children, and therefore interest in their mothers, is aligned with the global epidemiological transition, increasing the complexity of the profile of care given at the institute.

## Tecendo o Instituto Fernandes Figueira: situando memória, experiência e cuidado

O médico pediatra Antonio Fernandes Figueira é figura histórica, retratada em uma série de publicações como profissional dedicado e na vanguarda de seu tempo. Este artigo, no entanto, não tem por centro a personalidade do médico, mas se dedica ao Instituto Nacional de Saúde da Mulher, da Criança e do Adolescente Fernandes Figueira (IFF) documentado, “narrativizado” e tecido. Convidamos os leitores a seguir essa tríade. Primeiro, como foi documentado, por um lado, em uma dissertação de mestrado não publicada (De Seta, 1997), que explorou cinquenta anos de história institucional do IFF escutando informantes privilegiados pertencentes à primeira geração de trabalhadores, que chegou na década de 1940 e, por outro, em um relatório de 1973 (Brasil, 1973) que registra as atividades e os desafios do IFF nessa época. Segundo, como foi narrativizado por três trabalhadores que ingressaram nos anos 1980, cujas entrevistas compõem o acervo de uma pesquisa em atividade denominada “Memória como Linha de Cuidado”, conduzida pelas autoras. Terceiro, como foi tecido pelas autoras deste artigo com base no argumento de que o IFF se configura como espaço, acionando memórias com base nas experiências de cuidado desenvolvidas por seus trabalhadores. Os dois primeiros pilares da tríade comparecem como duas seções do artigo, e o terceiro é a linha que tece seu movimento dionisíaco.

O IFF se revela um espaço constituído a partir de documentos e da memória de trabalhadores, assumidos como narradores (Santos, 2006). Com relação ao conceito de espaço, ele “deve ser considerado como algo que participa igualmente da condição do social e do físico, um misto, um híbrido. Nesse sentido, não há significações independentes dos objetos” (p.56). Esse entendimento faz com que convidemos o leitor a compreender as tessituras entre documentos e narrativas como um entrelaçamento do cuidado com trajetórias e memórias que conformam experiências. Valorizando esse circuito, afirmamos que a experiência integra o mundo com intencionalidade, como “resultado da relação entre o homem e o mundo, entre o homem e o seu entorno ... uma espécie de corredor entre o sujeito e o objeto ... essas coisas não são apenas externas” (p.58). Os actantes podem ser pessoas, objetos ou lugares, e possuem a capacidade de ser significados, interpretados e ganhar sentido (Latour, 2000).

Inspiradas pela teoria ator-rede (Haraway, 1995; Law, Mol, 2009; Oliveira, 2016), situamos o IFF como um espaço (necessariamente articulado a temporalidade, intersubjetividade, intencionalidade e produção de sentido) onde interagem atores humanos e não humanos. Como o instituto não existe em abstrato, desvinculado dos encontros em sua espacialidade viva, constitui-se como lócus para “narrativizar” memórias. Esse exercício se encontra com aquilo que Ecléa Bosi (1994, p.458-459) define como “narrativa memorialista”, na qual o que importa é a tessitura entre a perspectiva pessoal dos acontecimentos e a maneira como lugares e pessoas se aconchegam no colo da memória narrativizada. As autoras deste artigo se colocaram como acolhedoras de memórias e foram elas próprias, como Bosi (1994, p.38), testemunhas no escutar, ouvir, registrar e transmitir lembranças, além de as buscar em documentos de outrora. Memórias não se bastam como lembranças. Memórias vinculadas às experiências de cuidado, articuladas a processos de “narrativização” a convite, traduzem a geografia hospitalar em espaço.

O que serve de linha nessa tessitura é o cuidado em acordo com Joan Tronto (1997, p.187-188), para quem “cuidar implica algum tipo de responsabilidade e compromisso contínuos ... Se cuidar envolve um compromisso, deverá, então, ter um objeto. Assim, cuidar é necessariamente relacional”. Assumindo essa definição relacional de cuidado intrinsecamente ligado a responsabilidade, compromisso, carga e moralidades, reconhecemos que o cuidado se materializa em práticas e narrativas, que conformam o que seria importante ou valioso para uma dada sociedade. O cuidado, portanto, remete a poder, às estruturas de desigualdade e opressão e a um campo de significados, expressos nas memórias vivas de quem as enuncia. O cuidado mundifica, cria mundos: “Tocar, considerar, devolver o olhar, devir-com ... tudo isso nos torna responsáveis pelas maneiras imprevisíveis nas quais os mundos tomam forma. No toque e no olhar, os parceiros, querendo ou não, estão na lama miscigenada que infunde nosso corpo com tudo o que trouxe esse contato à existência” (Haraway, 2022, p.58).

Para criar mundos precisamos simbolizá-los, e a narrativa se configura como a arte de contar histórias de novo, aninhando o processo de narrativização nas tradições de contar algo aos outros (Benjamin, 1994). Essa arte exige também um dom de ouvir, promovendo uma comunidade de ouvintes (p.205), como também Ecléa Bosi (1994) nos lembra em sua obra. Ao recuperar esse exercício de narrativizar, Benjamin nos convoca a recuar no tempo, compreendendo que um certo “dom narrativo” se entrelaça ao trabalho manual e ao encontro coletivo.

O cuidado pelo exercício relacional de transmissão alinhava um saber-fazer. Tais processos, que ocupam ainda um lugar secundário em relação aos objetos da ciência, se configuram nas conversas informais ou nas provocações à fala, como no caso das entrevistas de uma pesquisa. São justamente essas narrativas que acionam memórias e experiências que nos interessam. Na forma artesanal de comunicar se inserem as marcas do narrador tal qual, como nos diz Benjamin (1994), “a mão do oleiro na argila do vaso”. No encontro entre ouvinte e narrador se tece a memória e se inaugura a transmissão.

Como pesquisadoras engajadas em práticas de atenção a saúde, pesquisa e formação no IFF, assumimos o exercício de “memorializar” – fazer memória, não esquecer, rever processos dinâmicos de transformação do cuidado – enlaçado ao exercício de esperançar a saúde da criança, da mulher e do adolescente por mais cem anos.

## Uma espiral de gerações: a artesania metodológica

No imbricamento de documentos (De Seta, 1997; Brasil, 1973) e narrativas – advindas do acervo da pesquisa em andamento “Memória como Linha de Cuidado” – emerge o marcador geração como categoria analítica que opera articulada à de memória do IFF. Para Manheim (1952), a geração opera como um fenômeno social que categoriza e localiza socialmente os indivíduos, tal qual a classe social. Manheim afirma que “a unidade de gerações é constituída essencialmente através da similaridade de situação de vários indivíduos dentro de um todo social” (p.71). Nesse enfoque, a cronologia biológica com seus ritmos próprios (nascimento, envelhecimento, morte) se destacaria pela relevância sociológica atribuível a tais fenômenos, compreendendo a geração como um “tipo particular de situação social” (p.72), em que se compartilham sentimento, pensamento e comportamento.

Assim, “o ‘fenômeno da geração’ não representa mais que um tipo particular de identidade de situação, abrangendo ‘grupos etários’ relacionados, incrustados em um processo histórico-social” (Manheim, 1952, p.73; destaques no original). Para o autor, a distinção entre memória apropriada e adquirida está marcada pelo vínculo experiencial, não necessariamente presente na primeira. O que criaria uma situação comum – central para definição de geração – é estar em uma posição que permita experienciar os mesmos acontecimentos e dados, com semelhanças na forma de classificar essas experiências, o que não está definido pela contemporaneidade cronológica. A geração não se configura como idades cronológicas, mas pelo compartilhamento de tempos e experiências comuns ao articular nas narrativas transformações na atenção clínica prestada e no perfil de público que passa a compor o IFF. A geração que narra o IFF da década de 1940 remetia a um perfil de adoecimento de crianças mais relacionado à pobreza e à desnutrição, o que nos anos 2000 é substituído por tecnologias de suporte à vida, desde técnicas cirúrgicas intrauterinas até pediátricas, com recursos nutricionais especializados.

Esse acervo de documentos citados e narrativas escutadas evoca um testemunho público (Boltansky, 2015), remetendo a um *ethos* institucional e a um certo espírito de época. O *ethos* institucional do IFF conformou-se baseado em especialização, referência e sensibilidade para identificar a gravidade, que teve em Fernandes Figueira, médico pediatra e chefe do Departamento Nacional da Criança, seu patrono e nome de batismo institucional. Esses elementos fazem com que a noção de referência não seja apenas atribuída por “portarias ou normas”, mas por uma construção identitária, reconhecimento externo, atribuído tanto pela comunidade de especialistas quanto pelo público que busca atendimento em seu serviço materno-infantil.

### Sobre os narradores da pesquisa “Memória como Linha de Cuidado”

O projeto de pesquisa em andamento “Memória como Linha de Cuidado”, uma das fontes deste artigo, reuniu, entre fevereiro de 2021 e março de 2022, 29 narrativas de trabalhadores, de nível médio e superior, em atividade ou aposentados, com inserções diversas nas várias frentes de cuidado, seja em atividades-fim ou meio. A composição desse conjunto foi norteada principalmente por uma rede de informantes privilegiados.

Para este artigo, selecionamos três entrevistas emblemáticas porque localizam mudanças físicas na estrutura do IFF, ao mesmo tempo que resgatam transformações no público de crianças e adolescentes que passou a compor o perfil de referência na atenção pediátrica.

Defendemos que os três narradores, escolhidos entre as 29 entrevistas, possibilitam-nos identificar um olhar global do IFF a partir de suas atuações. São interpretações que articulam não simplesmente uma visão do setor onde atuam, mas uma visão que evoca a missão original materno-infantil (Brasil, 1973). Para nossos narradores – um médico de 60 anos, uma técnica de enfermagem de 66 anos e um administrador hospitalar de 56 anos – o espaço se organiza pelo cuidado com mulheres, crianças e adolescentes e na manutenção e defesa do IFF, de sua integridade. Isso implica que, mesmo na diversidade das carreiras escolhidas, há uma dedicação importante ao cotidiano do instituto. Se o médico (José Luiz de Carvalho, médico *staff*, 60 anos, em atividade na Unidade Intermediária do IFF, admitido em 1985) e a técnica de enfermagem (Vanilda Cipriano de Souza, técnica de enfermagem, 66 anos, aposentada, admitida em 1983 e aposentada em 2013) concentraram suas trajetórias na atenção clínica pediátrica, o administrador (Carlos Augusto de Andrade Meirelles, 56 anos, gestor da manutenção, em atividade, admitido em 1987) desenvolveu uma visão de conjunto e articulada de todo o instituto, compreendendo que sua atuação alcança o cuidado de quem ocupa as enfermarias e os ambulatórios. Reside na narrativa deste último uma complementaridade às duas outras narrativas. Outro critério para a escolha das três narrativas foi a convergência do início de suas trajetórias laborais no IFF na década de 1980, mediante seleções que seguiam as redes de sociabilidade, vínculos familiares, de conhecimento e amizade, e a busca por formação especializada.

Destacamos que o perfil de gravidade associado a cronicidade e complexidade do perfil de neonatos e crianças que passaram a ser encaminhados ao IFF como unidade especializada, a partir da década de 1980, exigiu que a atenção pediátrica se desdobrasse e demandasse atenção neonatal à prematuridade articulada à vertente obstétrica de alto risco fetal e de malformações. Os narradores presentes neste artigo “memorializam” transformações que remetem a uma transição epidemiológica na qual emerge uma “nova pediatria” (Moreira, Goldani, 2010).

Nossos entrevistados, como repositórios vivos de memória institucional, conjugam qualidades de narradores e de fontes históricas que transbordam suas amplas capacidades de recordação. Evocamos aqui dimensões de reflexividade, de exterioridade e proximidade crítica simultâneas que possibilitam o acesso a um plano maior de eventos que falam a respeito de uma época. Elas mundificam o cuidado, trazendo-o para a concretude do prosaico, do cotidiano, em uma teia de significados que interceptam pessoas, coisas, lugares, paredes, configurando espaços vitais e de poder.

Os nomes próprios dos narradores são mantidos, em concordância com a pesquisa – aprovada pelo Conselho de Ética em Pesquisa do IFF – e com a autorização expressa dos entrevistados.

### As memórias das décadas de 1940 e 1970

**Figura 1:**
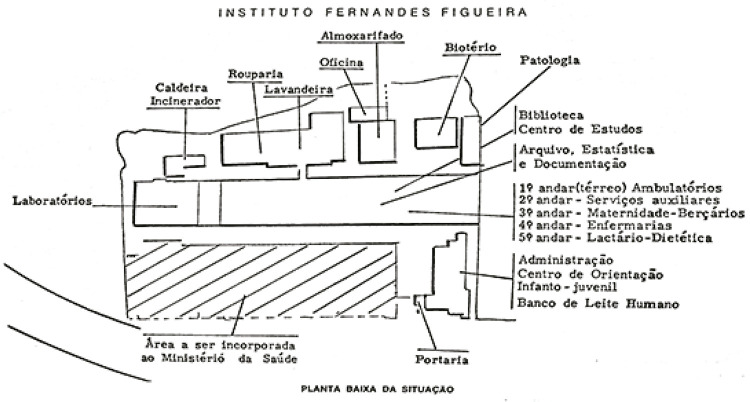
: Planta física do IFF exibida no relatório de atividades de 1973 (Brasil, 1973, p.132)

**Figura 2:**
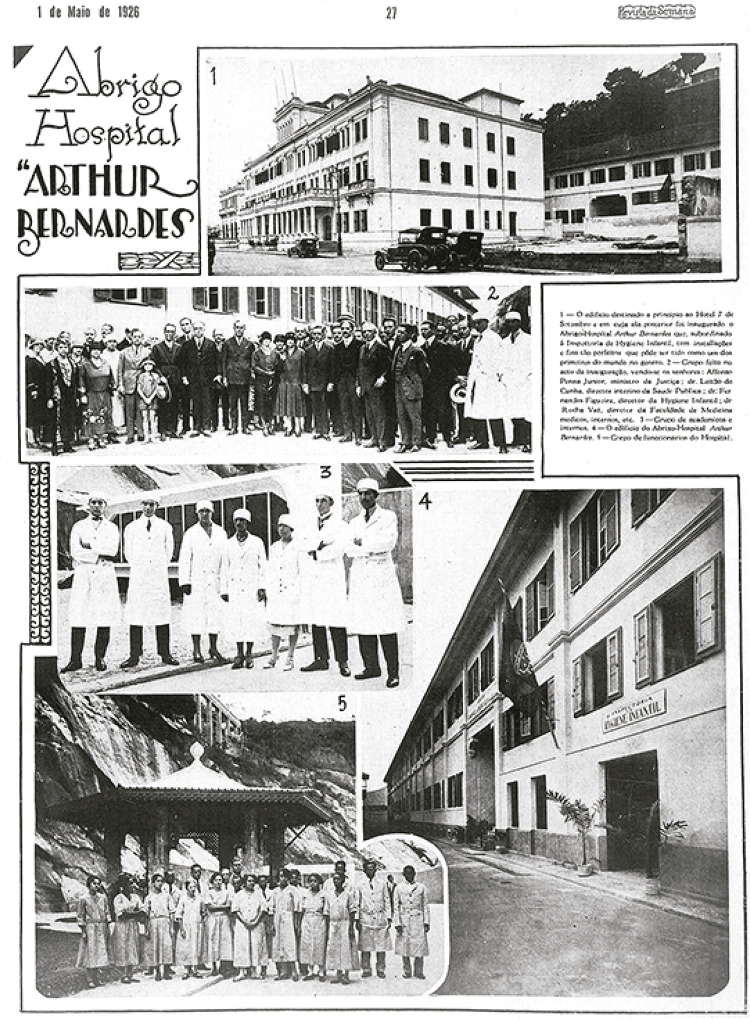
: Fotos do Abrigo Hospital Arthur Bernardes (*Revista da Semana*, 1 maio 1926, p.27)

**Figura 3:**
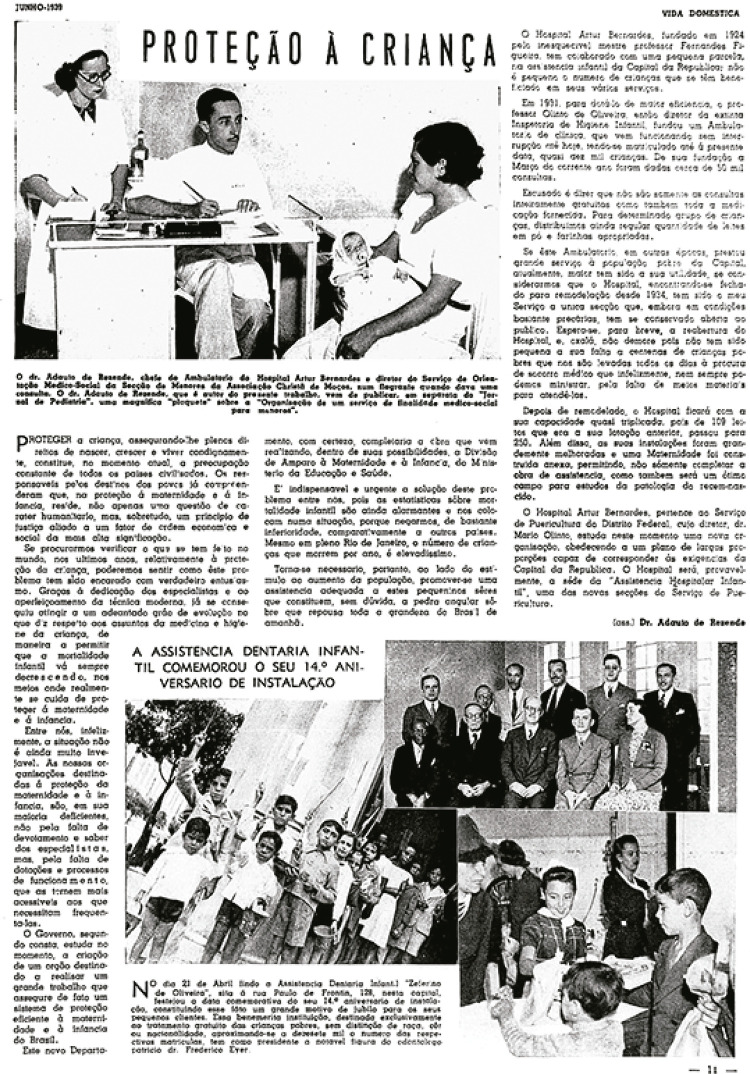
: Reportagem “Proteção à criança” narrando atividades e reforma do Hospital Arthur Bernardes (*Vida Doméstica*, jun. 1939)

A unidade da Fiocruz que desde 2010 se reconhece como Instituto Nacional de Saúde da Mulher, da Criança e do Adolescente Fernandes Figueira (Brasil, 21 dez. 2010) passou por transformações não somente no nome, mas também na planta física, na missão e no perfil (Figura 1). Nasce como Hospital Abrigo Arthur Bernardes (Figura 2), com “atividades de medicina geral e atividades de ensino e pesquisa” (Brasil, 1973, p.117). Passa a se chamar Instituto Fernandes Figueira pela lei n.4793, de 7 de janeiro de 1925 (Brasil, 1973, p.117), reconhecido como órgão de orientação de âmbito nacional na área materno-infantil (Figura 3). Com a extinção do Departamento Nacional da Criança e a sede do governo federal transferida para Brasília, é incorporado à Fundação Oswaldo Cruz em 1970. Segundo o relatório de atividades do IFF de 1973, o instituto teria como missão na época “realizar estudos e pesquisas sobre Maternidade, Infância, Adolescência e problemas sociais correlatos” (p.117).

O relatório de atividades de 1973 (Brasil, 1973) colabora para reunir uma história dispersa, que remete à personalidade do médico Antônio Fernandes Figueira. Com um olhar de vanguarda e protagonista na história da pediatria brasileira, defendeu, pela ciência, o direito das mulheres trabalhadoras à creche e à amamentação de suas crianças (Marques, 2018). Geograficamente, o IFF se localiza no bairro do Flamengo, zona Sul do município do Rio de Janeiro, na divisa com o Morro da Viúva e o antigo Hotel Sete de Setembro (Figura 2), primeira residência das enfermeiras em internato da Escola de Enfermagem Anna Nery (Moreira, 1999). O prédio Mario Olinto, com a estátua da mãe com seu filho, inclui hoje toda a área administrativa, direção e ensino; nas décadas de 1940 a 1970 comportava espaços de atenção de ginecologia, obstetrícia e o Centro de Orientação Juvenil. Como referência para atenção à saúde de mulheres, crianças e adolescentes no Sistema Único de Saúde (SUS), é reconhecido como instituto nacional (Brasil, 21 dez. 2010).

A narrativa desse relatório faz depreender uma tensão e muitas crises não somente relacionadas à precariedade estrutural do prédio do IFF, que ocupava os fundos do Hotel Sete de Setembro, mas também a cortes orçamentários que induziam o IFF a se tornar um “simples hospital assistencial” (Brasil, 1973, p.117). Consta ainda a ameaça de ser desvinculado da Fiocruz, quando os orçamentos não atenderem às necessidades da área de pesquisa do IFF (um instituto destinado à assistência médica como “atividade-meio” para atrair mães, crianças e adolescentes, isto é, o objeto de estudo, para a “atividade-fim”, que é a pesquisa (p.118). Os destaques são do próprio relatório e salientam um olhar entre objetos do conhecimento, atividades e missão. Naquela época já se defendia a incorporação da área do Hotel Sete de Setembro, pertencente à Escola de Enfermagem Anna Nery. Os argumentos para enfrentar as ameaças ao IFF incluíram a produção desse relatório com mais de 130 páginas e uma justificativa na forma de perguntas, caso o IFF fosse extinto ou resumido a um hospital assistencial: “Quem fará o estudo e a pesquisa Materno-infantil? As cátedras de pediatria das Faculdades de Medicina? Os hospitais infantis estaduais desprovidos de maternidade anexa?” (Brasil, 1973, p.114). O argumento segue remetendo à importância dos institutos como entidades que se fundamentam em pesquisa e formação de quadros.

Com relação à pesquisa de De Seta (1997), os dez informantes privilegiados entrevistados na ocasião eram testemunhas vivas das transformações e crises vividas pelo instituto, fazendo parte da geração que ali ingressou na década de 1940.

A autora resgata os desafios enfrentados para a consolidação do instituto, bem como da atenção à saúde de mulheres, crianças e adolescentes no Brasil. Constrói uma cronologia do instituto (De Seta, 1997, p.138) que permite o diálogo com Sanglard e Ferreira (2014) sobre as disputas e os desafios para o reconhecimento da pediatria como campo de especialidade, e da saúde materno-infantil como área de investimento.

De Seta (1997) dá destaque à valorização do serviço social em seu período inaugural. Esse setor foi constituído com o *status* de agência na década de 1940 e alocado no interior do então Instituto Nacional de Puericultura, subordinado ao Departamento Nacional da Criança, com recursos iniciais da Legião Brasileira de Assistência (LBA) (p.93). Essa agência ocupava todo o térreo do hoje Ambulatório Geral de Pediatria, o que, segundo a assistente social entrevistada por De Seta (1997, p.94), revelava sua importância. A ela cabia: trabalhar com a metodologia de casos; proceder a colocação em lares substitutos as crianças com alta hospitalar, mas cujas famílias tinham dificuldades ou cujas mães se encontravam internadas; e organizar a recreação das crianças internadas, sob o acompanhamento de auxiliares de enfermagem. Como nos lembra Silva (2018), a LBA, agência de Estado, tinha como função o amparo à maternidade e à infância, ratificando o texto constitucional de 1946. No período que vai da década de 1940 à de 1960, a mortalidade infantil era considerada resultado da negligência das mães. Nessa direção, ações destinadas ao núcleo materno-infantil estruturavam-se a partir de um olhar de responsabilização das mães pela saúde dos filhos, cuja ignorância deveria ser enfrentada pelo investimento na puericultura. Podemos então inferir que essa importância do serviço social dentro do IFF concorria com a medicalização da infância e maternidade aliada ao saber médico: “Médicos, higienistas e assistentes sociais passavam a ditar as regras para a medicalização e modernização da maternidade, criando a figura da ‘mãe-cientista’ para cuidar do recém-nascido” (Silva, 2018, p.1023; destaque no original).

De Seta (1997, p.34) destaca no depoimento de um médico puericultor três aspectos estratégicos da criação do Hospital Arthur Bernardes: (1) defender o binômio mãe-filho; (2) operar fora da administração direta das faculdades de medicina e ser referência para a saúde pública; (3) ensinar e formar profissionais de acordo com o conhecimento compartilhado entre pares, com pesquisas não necessariamente vinculadas à faculdade de medicina. Vê-se o lugar estratégico, e ao mesmo tempo sinuoso, que o então hospital ocupava. E o tal “binômio mãe-filho” atende ao que exploramos acima, reside no nome “Abrigo Hospital”, e se reúne na imagem da estátua que o simboliza. De acordo com o pensamento da puericultura higienista – que evoca a pretensão de construção da “maternidade científica” no Brasil (Freire, 2009) –, a presença da mãe era estratégica para que pudesse ser “educada” a realizar os cuidados adequados, ainda que não se deixasse de reconhecer também a sua importância na redução do estresse das crianças internadas. Havia, aliás, uma “Escola de Futuras Mães”, anexa ao hospital. A presença materna funcionava também como elemento organizador do espaço físico: no térreo, crianças desacompanhadas; no segundo andar, aquelas acompanhadas das mães (Freire, 2009, p.44).

A década de 1940 representou o apogeu do instituto, com pesquisas importantes em andamento, divulgadas pelo Centro de Estudos. Em 1949, o hospital fecha para obras (Freire, 2009, p.109), permanecendo o funcionamento do banco de leite e do ambulatório de pediatria no prédio Mario Olinto. O prestígio do Instituto Fernandes Figueira é fortalecido na década de 1950, mais precisamente em 1953, quando as dependências do instituto e do Centro de Estudos Olinto de Oliveira passam a ser utilizadas pela Sociedade de Pediatria. Ainda assim, esse apogeu científico e seu reconhecimento não foram acompanhados por melhorias na infraestrutura material, então de caráter precário.

Além da Agência Nacional de Serviço Social, com sede no instituto, o Centro de Orientação Juvenil foi a ele incorporado com a extinção do Departamento Nacional da Criança entre 1966 e 1969 (Freire, 2009, p.119). O Centro de Orientação Juvenil havia iniciado suas atividades em 1946 e, segundo Velloso (1956), tinha “como finalidade principal o estudo de técnicas de trabalho e o treinamento de pessoal no campo da ajuda psicológica aos adolescentes desajustados e seus responsáveis, ... desde 1946”.

O saber e a prática de assistentes sociais e psicólogos operavam afinados com o que era o pensamento social do Estado getulista, autoritário e centralizador, que tinha para a infância um projeto patriótico de base hierárquica, não diverso e positivista (Schmitz, Costa, 2017). A extinção do Departamento Nacional da Criança aprofundou uma crise com impacto na organização do instituto e na atenção prestada. Entre 1972 e 1977 as assistentes sociais eram responsáveis por avaliar, por meio de uma classificação socioeconômica, quem poderia ou não pagar pelos serviços oferecidos (p.122).

A leitura da pesquisa de De Seta (1997) apresenta localização memorial do IFF como espaço estratégico para a formação do Estado brasileiro, no olhar para crianças e mulheres. E o adolescente – muito ausente no curso do texto – comparece qualificado como o “juvenil”, evocando a visão corretiva do “desajustado”. Conforme Jacó-Vilela et al. (2017, p.93), o Centro de Orientação Juvenil inseria-se na “preocupação do governo estadonovista com a educação e assistência à criança e juventude como forma de ‘cuidar’ do futuro da nação”.

## Trânsitos das memórias da geração de 1980 e 1990 ao século XXI

Convidamos os leitores e leitoras a seguir conosco narradores que fazem parte da geração que chega ao IFF nos anos 1980 e relatam sua transição para os anos 2000. Nossos narradores compartilham: o prazer de contar; o impulso de testemunhar para o grupo que os convida/escuta/grava; a recordação de forma reflexiva sobre si e sobre uma trajetória compartilhada em que nomes, funções e espaços do IFF são também ressignificados. No aporte interpretativo das memórias e experiências narrativizadas, o hospital se delimita como um espaço por excelência para explorarmos a teoria ator-rede (Latour, 2000). Na associação entre atores humanos e não humanos, essa teoria reconhece o estabelecimento de conexões heterogêneas e inovadoras que cocriam e ampliam a concepção do social.

As crianças e suas mães mencionadas no relatório de 1973 (Brasil, 1973) e na pesquisa de De Seta (1997) são outras nesse espaço de trânsito do século XX para o XXI. Em consonância com a teoria ator-rede, o espaço interacional se renova no diálogo entre gerações e atores diferentes, fazendo-nos compreender que interpretações, práticas, interesses e ações se reconfiguram distintamente ao longo do tempo e do espaço de acordo com o contexto vigente. Vanilda – técnica de enfermagem de 66 anos, já aposentada – e José Luiz – médico pediatra de 60 anos – retratam, em suas narrativas, quem era a criança atendida no IFF nos anos 1980.

Eu cheguei [1983], vim de casa de saúde particular, trabalhava com crianças bonitas, cheirosas, com pais educados. No Fernandes Figueira, nessa época, as crianças não ficavam acompanhadas, não tinha Estatuto da Criança ainda. As crianças ficavam sozinhas e eram crianças muito carentes, muito pobres, muito sujas. Tinha um convênio que se chamava Febem. ... Principalmente nos anos 80 no Brasil, eu sabia da pobreza, da miséria, mas eu não convivia com ela diretamente e aquilo me deixava chocada. O perfil da instituição na época em que eu entrei ... a criança era só uma coisa que você tinha que cuidar (Vanilda Cipriano de Souza).

Essa imagem de que a criança “era só uma coisa que você tinha que cuidar” evoca um certo espírito de época em que a construção da ciência operava sobrepondo razão a emoção (Freire, 2009). O cuidado entendido como relacional e materialmente conformado (Tronto, 1997; Haraway, 1995) seguia essas prescrições, reservando à criança um lugar de objeto, e não de sujeito.

A relação do cuidado como campo de interdependências social e historicamente situadas nos faz compreender que a imagem pública que uma instituição como a Fiocruz evoca gera movimentos, por exemplo, de busca por formação.

Cheguei em 85 como residente de Pediatria. Era um hospital ligado a uma grande instituição, tem um peso bacana porque a gente sabia que é um local sempre de aprendizado. O perfil de pacientes era totalmente diferente do que a gente tem hoje. Naquela época a gente ainda trabalhava muito com a questão da desnutrição, que era uma realidade muito grande. A gente fazia atendimento a crianças institucionalizadas. Existia um grande abrigo de crianças aqui perto, então a gente fazia atendimento de urgência basicamente para essa população. Tinha muito óbito, muita morte não explicada, não esperada. Porque a gente monitorizava menos. Como a gente lidava com desnutrido, era sempre muito risco. Com a melhoria da condição social, as doenças começaram a se transformar. Então a gente passa a ter esse perfil de crianças complexas crônicas (José Luiz de Carvalho).

Cabe dizer que essa transformação de perfil referida, com a nomeação de crianças complexas crônicas que não são mais as desnutridas, destaca-se como evento marcante, que recoloca o IFF na cena nacional. Essa classificação – condição médica complexa em pediatria, ou crianças com condições de saúde crônico-complexas – já era utilizada no âmbito internacional desde meados da década de 1990, e, a partir de uma pesquisa que traçou o Perfil de Internações Pediátricas na Enfermaria de Pediatria do IFF/Fiocruz em 2014 e 2015, ganhou visibilidade interna e externa. Esse campo de definições remete às crianças com morbidades de diversas ordens, incluídas em quadros de causa genética, inflamatória, ou adquirida que comprometem diversos sistemas, levam a dependência de tecnologias diversas, internações prolongadas, e que são nomeadas como condições médicas complexas, condições crônicas complexas de saúde. A recomendação após a pesquisa ter sido conduzida é de que a melhor conceituação para definir as necessidades dessas crianças remete à cronicidade e à complexidade de suas condições de saúde, que podem nos levar a planejar e dimensionar custos, investimentos, tratamentos e linhas de cuidado intersetoriais e integrados para seu maior benefício (Moreira et al., 2017).

Essas transformações no perfil das crianças atendidas nos levam a reconfigurar os sentidos de infância que a Constituição brasileira de 1988 e o curso da década de 1990 fazem emergir, entre eles a criança e o adolescente como sujeitos de direitos. Tal reconfiguração nos faz compreender o sentido da infância como uma construção histórica e social (Pires, 2008, p.138): “Infância é uma variável da análise social. Ela não pode nunca ser separada das outras variáveis, como classe, gênero ou etnicidade. Análises comparativas e interculturais revelam uma variedade de infâncias, e não um fenômeno único e universal”. Ao mesmo tempo, outro perfil de adoecimento, de demandas das crianças por cuidados de saúde, promove uma transformação da assistência e da própria instituição. Transformam-se espacialidades, formações profissionais, relações e tecnologias de cuidado.

Ainda sobre o perfil de infância que era configurado pelas doenças da pobreza, havia a construção de um saber fazer, base do cuidado institucional, no qual:

Eu nunca recebi orientação diretamente, ‘Não, você não pode botar criança no colo para dar mamar’. Não, nunca recebi essa orientação, mas quando eu cheguei esse era o método que era usado. Você está chegando num lugar, num emprego que você precisa trabalhar, então você tem que seguir a cartilha para não ser diferente. E quando eu tentava ser diferente, eu era criticada. ... A criança estava ali para ser cuidada, dar banho, dar comida e botar na cama. E pronto (Vanilda Cipriano de Souza).

Essas formas de fazer, sem precisar ser ensinadas, operam como substância do cuidado (Tronto, 1997) e configuram uma cultura e sua espacialidade. O espaço (Santos, 2006) expressa as interações, distribuições de interesse e afetos, bem como as relações com a sociedade em suas desigualdades, distribuição de poder e opressões. Ao acionar suas memórias, a informante nos apresenta com alguma frequência as expressões “perfil” e “feudo”, demarcando uma planta sociotécnica do instituto a partir da distribuição de poder.

Eu era da quinta enfermaria, (que) era Clínica Geral e Desnutrição. Era chamada a enfermaria da ‘M’. Porque a gente fazia uma pesquisa para a Fiocruz na época, que tinha que botar a criança no peniquinho, e a criança tinha que fazer cocô e você tinha que juntar aquele cocô e botar num vidrinho e enviava para a fundação. Eram crianças pequenas, na faixa de 2, 3 anos, e bebês. Esse era o perfil das crianças. Muitas vezes na madrugada, doutor Zé Luís, residente, via eu correndo de um lado pro outro igual uma barata tonta: criança para nebulizar, criança suja, criança chorando com fome, criança chorando de solidão. E quantas vezes ele deu mamadeira para as crianças na madrugada, trocava fralda de pano, nebulizava (Vanilda Cipriano de Souza).

A presença de José Luiz é evocada nas memórias afetivas de Vanilda como médico que cuidava, ou, nas palavras do próprio, médico-enfermeiro. Essa leitura do cuidado relacionado ao oferecimento de conforto, suporte físico, atendimento às necessidades básicas da criança foi determinante para reificar cuidado como algo simples, de característica feminina, ligado ao afeto e, portanto, hierarquicamente menos valorizado como trabalho e profissão (Guimarães, Hirata, 2020).

Esse perfil da quinta enfermaria contrastava com o de outras, que organizavam a chamada atenção pediátrica de internação.

Primeira enfermaria era a de cirurgia, era primeiro mundo, como se tivesse uma barreira lá na frente da porta. Para cá tinha as outras enfermarias. Acho que tinha a segunda enfermaria, que era onde fazia os testes daquelas crianças da fibrose cística, tinha a quarta enfermaria. Eu achava, assim, cada enfermaria era um feudo de alguém (Vanilda Cipriano de Souza).

Carlos Meirelles incrementa a visão do hospital sob ângulos diferentes, localizando-o em um espaço que transcende seus muros ou setores.

Na época estava naquela transição ainda do regime militar, então o pessoal ainda tinha muito aquela disciplina do serviço público, dos horários. Eu cheguei aqui em 87, e o hospital sofreu várias transformações. Na verdade, esse espaço aqui mesmo que a gente está [corredor do atual Centro de Estudos], era um setor de triagem da Pediatria. Aquele espaço que hoje é o ambulatório de Pediatria, ele era só um corredorzinho de mais ou menos um metro e meio, com várias portas dos dois lados até lá no final, e o anfiteatro era lá perto do elevador de carga. Eu me lembro que no sexto andar tinha o CTI. Onde é o ensino hoje, era o COJ. Eu participei da implantação da biblioteca, da creche. A sala de parto era lá no meio do prédio, mais ou menos onde é a enfermaria de gestante. O terceiro andar era enfermaria. O berçário continua, o trecho é o mesmo. Foi de 87 em diante, ele [Paulo Roberto Boechat, ex-diretor] ficou 8 anos. Aí foi o ano que ele fez mais transformações no hospital. Na Direção, ele investiu em equipamento, em infraestrutura, investiu em contratação de pessoal de manutenção, de obras. Na Pediatria, no passado, no segundo andar, eram duas carreiras de banco, uma do lado de lá e outra de cá, e você passava no meiozinho com um montão de paciente no meio. Aí o médico abria a porta ali, tudo bem pertinho, e chamava o paciente (Carlos Augusto de Andrade Meirelles).

O trabalho desenvolvido no hospital estabelece uma orientação teleológica com o cuidado. No caso de Meirelles, ter construído uma trajetória no IFF junto ao Serviço de Manutenção permitiu uma visão de conjunto da instituição. Em sua narrativização, aponta a revisão das intervenções na planta física da organização ao mesmo tempo que nomeia uma infinidade de pessoas, como que para garantir que seus nomes fiquem registrados. Meirelles testemunha e encarna a construção de um lugar de confiabilidade entrelaçado a um projeto de instituição, engendrado fortemente pela agência de atores humanos e não humanos: as paredes e corredores, incubadoras e leitos, os cilindros de oxigênio. O narrador mobiliza afetos apresentando a produção de ar medicinal e a interação com os fornecedores, os geradores de energia e sua relação com a estação da Light, sem perder o foco no cuidado às crianças “ligadas nas máquinas” no Centro Cirúrgico e na UTI. “O Fernandes Figueira se tornou uma vida para mim. O instituto foi também moldando a gente com isso, da gente gostar de querer fazer as coisas em prol do instituto” (Carlos Augusto de Andrade Meirelles).

As relações de vizinhança com prédios de classe média alta do quarteirão da avenida Rui Barbosa, no Flamengo, são evocadas também nessa movimentação entre o lugar do IFF e seus projetos de cuidado. As relações entre “dentro” e “fora” são exemplarmente narradas por Meirelles. O projeto de cuidado, com suas rotinas operacionais, também afeta quem dele se avizinha sem o conhecer na intimidade. Na autoridade moral de quem o conhece, o IFF, personificado, como um actante, é apresentado por nosso narrador com sua imensa responsabilidade pública de sustentar e qualificar a vida de quem, por um tempo, também se torna um Fernandes Figueira. O excerto a seguir refere-se a um incidente no qual uma autoridade do Judiciário, moradora da vizinhança, muro a muro com o instituto, sentiu-se prejudicada pela rotina de abastecimento dos gases medicinais. Esse questionamento se desdobrou em ameaças e presença física daquela autoridade, acompanhada de policiais federais para interromper tal rotina, acionando por meio de Meirelles – identificado como o obstáculo – a direção do instituto. O diretor ratifica toda confiança e autoridade a Meirelles, desconstruindo a queixa. O mais interessante é o desdobramento da cena narrada apresentar ao operador da Justiça – ocupado com sua autoridade e interesse pessoal – o projeto de cuidado das crianças internadas. Esse projeto, que se configura coletivo, não está restrito à assistência direta de profissionais de saúde, mas também é operado pelos trabalhadores ditos “dos bastidores”, como os da área de manutenção e engenharia.

‘O meu nome é Meirelles, sou o responsável desse caminhão. Mas o quê que o senhor quer?’, ‘Eu quero que você pare o abastecimento!’. Eu falei: ‘Doutor, eu não posso parar. Esse é um sistema automático. Eu tenho vários pacientes pendurados na rede de oxigênio. Olha, para o senhor ter uma ideia, vou levar o senhor para conhecer nossos pacientes’. Levei na UPG [CTI do instituto], levei na neonatologia... (Carlos Augusto de Andrade Meirelles).

Nesse excerto de narrativa, a enunciação de poder e autoridade entre quem está fora do IFF e quem o conhece por dentro revela o compromisso com o cuidado. A discussão coloca em cena a instituição como um actante. Ressalta-se um projeto coletivo e a responsabilidade técnica de fazer o cuidado acontecer. O IFF se configura diferentemente para o “operador da Justiça e morador vizinho” e para o “chefe da manutenção e trabalhador da instituição”. O actante Instituto Fernandes Figueira, em configurações de significado distintas, opera em circuitos de redes entre atores humanos e não humanos, que dão visibilidade ou encobrem a existência das crianças. Para o “operador da Justiça e morador vizinho”, o instituto resume-se a um barulho que perturba o “silêncio da vizinhança”, ou ao muro que faz divisa entre seu prédio e o instituto e precisa de manutenção. Já para o “chefe da manutenção e trabalhador da instituição”, o instituto é exatamente o lugar de cuidar de crianças que, para ser reconhecidas em sua existência, precisam ser apresentadas.

Há nessa rede sociotécnica a circulação de autoridade, conhecimento, responsabilidade e vínculos, remetendo a muitas particularidades da história do instituto. Uma delas recua à época na qual o ingresso no IFF muitas vezes acontecia por indicação de um parente consanguíneo, conhecido ou amigo, ou por ter sido egresso do Programa de Residência. Entrar em uma instituição significa construir uma trajetória de reconhecimentos que, na época, não contava com o concurso público como exigência.

Eu vim por causa do meu pai. Meu pai trabalhava na cozinha e depois foi trabalhar no CME (Central de Esterilização) (Carlos Augusto de Andrade Meirelles).

Eu trabalhava numa casa de saúde na Tijuca. E uma noite de plantão lá estava a doutora Lúcia [Lúcia Monteiro, cirurgiã pediátrica]. Ela me falou: ‘Vanilda, a Fundação Oswaldo Cruz está abrindo inscrição para auxiliar de enfermagem’. Aí cheguei lá, me inscrevi, o departamento pessoal me aplicou uma provinha. Recebi um telegrama, não sei, dois, três dias depois, mandando eu me apresentar com uniforme para trabalhar (Vanilda Cipriano de Souza).

Meirelles, quando fala dos funcionários do seu setor, refere-se à substituição progressiva ao longo dos anos dos vínculos trabalhistas vigentes na época por outros mais frágeis, instáveis e terceirizados, modificando a vinculação ao trabalho e o próprio sentimento de pertencimento ao grupo.

Configura-se ainda, entre as versões do instituto narrativizado, a transformação da planta física:

A grande mudança que teve no IFF, para mim, foi nos anos 90. De 83 até os anos 90, eu trabalhei com o mesmo sentimento, a mesma esperança de mudar, e mudou para melhor. Entraram enfermeiras novas, entraram os médicos novos, mudou o perfil das crianças, que para mim não foi uma coisa que eu gostei. Porque eu gostava da criança que falava, que brincava, que conversava, que me chamava de tia. Isso me fazia feliz. A partir dos anos 90, se eu não estou enganada, teve uma reforma no hospital. Porque nos corredores do hospital não tinham janelas. Era tudo vidro quebrado, entrava um frio desgraçado na enfermaria. Não tinha fralda descartável, a gente usava fralda de pano nas crianças. Então tinha dia que era um desespero tão grande você ter aquela criança com diarreia e você não ter uma fralda para botar (Vanilda Cipriano de Souza).

Cabe ressaltar que essas transformações na planta física acompanharam uma complexificação no perfil das especialidades que remetem à discussão do *ethos* profissional que fizemos anteriormente, e em sinergia com as necessidades de crianças e gestantes que chegavam ao IFF com perfil de risco fetal e neonatal. O IFF é reconhecido também pela atenção de referência em pesquisa, diagnósticos e cuidados na área de genética, desde a década de 1970 (Brasil, 1973), para diagnósticos pré-natais. Essas narrativas podem ser lidas à luz do que Moreira e Goldani (2010) denominam “nova pediatria”, com melhoria nos indicadores sociais globais, ainda com distribuição desigual de renda e acesso a saúde, saneamento e direitos humanos. Na época referida por Vanilda, conquistávamos no Brasil a redução das taxas de mortalidade infantil entre 2 meses e 5 anos de vida em virtude de um bem-sucedido Programa Nacional de Imunizações, com concomitante investimento no aleitamento materno apoiado. Acompanha esse cenário as conquistas no campo da engenharia biomédica, conhecimentos e técnicas de cirurgia pediátrica, reabilitação funcional na área de fisioterapia motora e respiratória, ações de enfermagem relacionadas ao manejo de cateteres e suas manutenções, nutrição enteral e parenteral acompanhada de ações no campo da fonoaudiologia hospitalar e de linguagem (Moreira et al., 2017). Ainda, as transformações no campo do conhecimento relacional, de abordagem e manejo da clínica, da criança como sujeito de direitos também representaram conquistas no campo da atenção psicológica, de serviço social, terapia ocupacional, cuidados paliativos e apoio de um voluntariado organizado. Os artigos de Moreira e Goldani (2010) e Moreira et al. (2017) contribuíram para o debate, reconhecimento e visibilidade de conceitos que já circulavam internacionalmente como condição crônica complexa pediátrica (Feudtner et al., 2001), crianças com necessidades de cuidado especial (McPherson et al., 1998), criança com condição crônica (Simon et al., 2010) ou ainda crianças com complexidade médica (Cohen et al., 2011).

Abriu a Pediatria novamente depois da reforma. Isso acho que já depois dos anos 90, se eu não me engano. Chegaram funcionários novos, enfermeiros e médicos novos. Foi um período difícil de adaptação, porque a gente vinha de um rameirão na velha Pediatria. E chegaram novas tecnologias, chegaram bombas infusoras. Eu lembro que a funcionária da empresa foi lá, ensinou a enfermeira do dia e pronto. Aí eu falava com a minha colega: ‘Mas isso não é possível! Como eu vou trabalhar com essa bomba se eu não sei, se ninguém me explicou?’. Então aprendemos a trabalhar com a bomba infusora sozinhas. A nova Pediatria, com a nova realidade, foi sofrida (Vanilda Cipriano de Souza).

Muda porque a gente está vivendo um caos social gigante nesse país há anos, um caos político-econômico-social. Então as famílias se transformaram, não são mais talvez tão presentes, como eram. E mudou o doente, o perfil mudou muito. O que antes morria por falta de abordagem, de espaços, de entendimento, hoje em dia a gente prolonga muito mais tudo isso. As crianças que morriam no primeiro ano de vida por insuficiência respiratória, hipertensão pulmonar, estão tendo alta em condições razoáveis (José Luiz de Carvalho).

Na intitulada “nova pediatria”, o perfil se distancia das crianças típicas, já que os corpos expressam diferenças que desafiam normas de mobilidade e estética. Na cena narrada a seguir, fica flagrante como um médico chegado no concurso dos anos 2000 se surpreende e define as crianças de que passará a cuidar:

Aí ele falou pra mim: ‘Quando eu vim conhecer a UI de dia, cheguei em casa e falei para a minha mulher: Fulana, a criança mais bonita que tem na UI (unidade intermediária) não tem um olho’. Um com tubo na boca, outro com um tubo no nariz, outro com negócio saindo aqui do lado. Não tem mais criança normal, criança que corre, não tem mais criança assim. Então quando mudou, quando virou para essas crianças, Ave Maria, foi tão difícil! (Vanilda Cipriano de Souza).

Cabe ressaltar que a atenção pediátrica muda porque passamos a ter uma complexificação do conjunto do perfil do IFF com outras portas de entrada, como da genética articulada à medicina fetal, a cirurgia pediátrica para correção das malformações em crianças nascidas no IFF e fora dele, a criação da neonatologia como um departamento, com atenção aos prematuros de muito baixo peso, e daqueles que passam a construir itinerários no IFF desde a obstetrícia até a pediatria. No relatório de 1973 (Brasil, 1973) se faz menção aos neonatos “anormais”, mas não com uma atenção vinculada à neonatologia, que é inaugurada no IFF em 1985, depois reformada em 1987.

Faz parte dessa transição entre uma “velha” e uma “nova pediatria” o que podemos denominar uma “espacialização estendida”. As fronteiras do cuidado se alargam e incorporam as casas que essas crianças da nova pediatria passarão a conhecer tardiamente, já que se tornam moradoras do Instituto e requerem um processo complexo de desospitalização:

‘Meirelles, a gente não tem condições de levar o Bipap (ventilador mecânico portátil), levar o monitor, está arriscado a ter um curto-circuito, porque a instalação é toda precária. Tem como você fazer?’. Falei: ‘Vou lá dar uma olhada. Se for possível, a gente faz’. Fomos lá, fizemos toda a instalação da casa. E assim a gente acaba, ao longo do tempo, se envolvendo também com as questões das próprias crianças, da família da criança, as questões sociais dela. Lá como ela vive, o local, estrutura de casa, a gente dá opinião. Eu já fui em vários lugares, até já fomos em comunidade (Carlos Augusto de Andrade Meirelles).

Faz parte do cuidado estendido essa organização de redes sociotécnicas nas quais a ação de voluntários organizados pelo Núcleo de Apoio a Projetos Educacionais e Culturais se reúne a um diagnóstico de necessidades das crianças. A articulação com a equipe interdisciplinar da desospitalização procura garantir às crianças o direito de viver em casa, viabilizando esse projeto.

Andou porque a gente criou dentro do serviço um projeto para tentar facilitar a saída dessas crianças, já que a gente não conseguia lidar com a coisa pública, com o Estado auxiliando. Nem as famílias com condição de ter alguém dentro de casa em sistema de *home care*, nem o Estado conseguia prover tudo isso. Então o projeto de desospitalização é interdisciplinar. A gente agora começa a tratar pensando já numa possibilidade de desospitalização, coisas que a gente não tinha há dez anos. Era muito difícil, muito doído (José Luiz de Carvalho).

As narrativas apontam que o qualificativo “complexo” não se reporta apenas à definição de complexidade clínica, mas abrange também o cuidado compreendido materialmente, relacionado às condições de vida e desigualdades sociais. O cuidado remete às figuras femininas, expostas a processos ativos de precarização e vulnerabilização da vida. A complexidade tem gênero, raça e território. E, nessa direção, mesmo antes da chamada mudança de perfil, já havia um movimento de referir os cuidados das crianças para locais próximos de onde moravam. As moradias, entretanto, não apresentavam em geral a estrutura necessária:

Tinha uma necessidade de mandar ela [a criança que há três anos morava no Instituto] para perto de casa. Já estava entrando naquele programa de tentar organizar uma maneira de mandar para perto de casa. E ela morava numa casa de pau a pique, fogão de lenha. Você entrava na casa, em dois minutos saía preta de fuligem. Como uma criança com problema pulmonar grave ia para esse lugar? Não tem condição nenhuma de botar, na época, uma bala de oxigênio naquela casa com aquela situação (Vanilda Cipriano de Souza).

A legitimidade e virtude de reconhecer que as crianças precisavam se tratar perto de casa contrastava com um cenário em que o SUS, no marco da Constituição federal de 1988, engatinhava. Cuidar de crianças significa, como destacam Forrest et al. (1997), considerar cinco “Ds”: (1) desenvolvimento cognitivo, emocional, global como inerente à criança, e precisando ser garantido; (2) dependência de um adulto de referência que lhe ofereça cuidado e atenção à saúde; (3) diferenças que as distinguem dos adultos no que diz respeito ao desenvolvimento das doenças, riscos de agravos, vulnerabilidades nas próprias ações de cuidado; (4) demografia, que considera as condições materiais, econômicas e sociais em que vivem e que interferem diretamente no acesso aos cuidados, articulados aos “Ds” anteriores; (5) desigualdades relacionadas e que interferem nos quatro “Ds” anteriores e nos reenviam ao fato de que sobre as localizações sociais (raça, gênero, território) incidem as opressões e discriminações.

Outro destaque a ser analisado diz respeito aos tensionamentos e fricções entre mulheres diferenciadamente situadas na cena do cuidado hospitalar: mulheres da equipe de enfermagem e as mulheres mães das crianças.

Nessa época a mãe não podia ficar, antes do Estatuto (da Criança e do Adolescente) a mãe não ficava. Então ele [uma certa criança internada] ficava de sapato. Ninguém tira o sapato do pé dele. Ele dormia de sapato, era porque no dia seguinte ele ia embora. Se a gente dava banho nele, tinha que calçar o sapato. Ele ficava deitado no soro de sapato (Vanilda Cipriano de Souza).

A fricção está na cena da criança sempre calçando o sapato após o banho – um movimento de esperança feita prontidão para ir embora com a mãe. Quando a mãe visitava e não permanecia, a equipe compartilhava a tristeza da criança, mas também sentia alívio:

Depois que as mães passaram a ficar na enfermaria foi um grande ganho para as crianças. Bem grande, quem fez isso realmente vai pro céu, assim, direto. Mas para nós, do lado de cá, foi um tormento. Até a gente se adaptar com as mães, foi difícil. Porque as mães são muito difíceis, são pobres, são desalentadas da vida, filho doente, filho pequeno. Agora imagine você ter que entender 20 mães, que na enfermaria tinha 20 crianças. E você tendo que lidar com elas, com os filhos delas, com a doença dos filhos delas (Vanilda Cipriano de Souza).

A criança e o adolescente como sujeitos de direitos se concretizam no Estatuto da Criança e do Adolescente de 1990, em cujo esteio se reconhece o direito ao acompanhante. Na velha pediatria, a solidariedade pessoal e local operava, por exemplo, para arregimentar o dinheiro de passagem para mães que tinham filhos longamente internados. Na nova pediatria, a presença das mães provoca tensões e sobrecargas emocionais, o que poderíamos chamar de um ônus do direito ao cuidado.

Eu sonhei que eu saía de manhã e não existia mais nada, era tudo uma névoa. Aí eu pensava: ‘Meu Deus, o mundo se acabou! Agora eu vou ter que viver, passar o resto da minha vida aqui dentro desse hospital?’. E eu virava de costas e entrava para o hospital, e acordei. Falei: ‘Vou no Departamento Pessoal pedir minha aposentadoria hoje!’ [risos]. Porque isso é o fim [risos] da minha existência. E assim foi feito. Tá bom? (Vanilda Cipriano de Souza).

Memórias significativas se forjam pelas emoções a elas vinculadas. O sonho narrado por Vanilda foi vivido em um dia em que o Rio de Janeiro foi tomado por um de seus clássicos temporais. No mesmo dia, um médico muito querido morreu atropelado em frente ao Instituto.

## Considerações finais

Nestas páginas apresentamos o Instituto Nacional de Saúde da Mulher, da Criança e do Adolescente Fernandes Figueira a partir da memória tecida nas gerações, e que se torna fundamental para sua consolidação institucional. Cabe dizer que o fio narrativo aciona fortemente o cuidado com as crianças. Reconhecemos a pediatria como um articulador da imagem da criança a da maternidade, ou seja, a mulher no seu componente materno-infantil, tanto no relatório de 1993 quanto na pesquisa de De Seta (1997) e nas narrativas de nossos narradores. A criança e sua imagem no colo de sua mãe – símbolo retratado na estátua à frente do IFF e em seus logotipos oficiais – comparecem como fortes mobilizadores de afetos e memórias de cuidado que remetem ao seu patrono, o pediatra Antonio Fernandes Figueira. É pelo compromisso e relacionalidade do cuidado que se dá o reconhecimento do IFF na cena pública, interligando mulheres e crianças.

Sem qualquer pretensão de esgotar essa história, reconhecemos tratar-se apenas de uma versão, entre tantas outras possíveis, na apreciação desse espaço relacional. Cabe a ampliação de exploração desse universo em demais pesquisas que acionem outros personagens e/ou aprofundem aspectos específicos não alcançados aqui.

A perspectiva relacional do cuidado, na análise narrativa do acervo empírico e dos documentos mobilizados, articula, tanto quanto possível, a análise de atores humanos e não humanos na dinâmica de forças, fluxos e interesses.

A pobreza medicalizada e a tarefa de “corrigir” mães foi substituída pelo trabalho com crianças que desafiam todas as nossas concepções do que seja infância. Inseridas em um hospital onde a competência técnica e a especificidade dos casos clínicos são reconhecidas, acionar a dimensão narrativa significa apostar na dinâmica relacional como transformadora de afetos e do ato de cuidar. Quem trabalha ou é cuidado no IFF ganha a identidade Fernandes Figueira como um sobrenome.

Não é a geografia do Instituto que está em jogo sozinha. É como essa fisicalidade se traduz em enredos, em redes de conhecimento, disputas, lugares de onde se fala e se distribui o poder. Um poder justificado, que se volta para aqueles para quem, afinal de contas, existe o Instituto Fernandes Figueira: mulheres, crianças e adolescentes, além de seus trabalhadores e de quem chega até ele para formação.

## Data Availability

Não estão em repositório.
